# Analyzing Parental Involvement in Youth Basketball

**DOI:** 10.3390/sports12120350

**Published:** 2024-12-18

**Authors:** Maria V. Lopes, Andreas Ihle, Élvio Rúbio Gouveia, Adilson Marques, Cíntia França

**Affiliations:** 1 Department of Physical Education and Sport, University of Madeira, 9020-105 Funchal, Portugal; mariavarandaslopes@gmail.com (M.V.L.); erubiog@staff.uma.pt (É.R.G.); 2 Department of Psychology, University of Geneva, 1227 Carouge, Switzerland; andreas.ihle@unige.ch; 3 Center for the Interdisciplinary Study of Gerontology and Vulnerability, University of Geneva, 1227 Carouge, Switzerland; 4 Swiss Center of Expertise in Life Course Research LIVES, 1227 Carouge, Switzerland; 5 Laboratory for Robotics and Engineering Systems (LARSYS), Interactive Technologies Institute, 9020-105 Funchal, Portugal; 6 Interdisciplinary Center for the Study of Human Performance (CIPER), Faculty of Human Kinetics, University of Lisbon, 1495-751 Lisbon, Portugal; amarques@fmh.ulisboa.pt; 7 Environmental Health Institute (ISAMB), Faculty of Medicine, University of Lisbon, 1649-020 Lisbon, Portugal

**Keywords:** parents, athletes, boys, girls, fathers, mothers, sports

## Abstract

Parental involvement in youth sports is increasingly relevant in scientific research since parents have gradually developed awareness regarding youngsters’ sports experiences. This study aimed to (1) examine potential differences in athletes’ and parents’ perceptions of parental involvement practices in youth basketball, (2) verify whether these perceptions differ by sex, and (3) analyze the relationship between the different perceptions dimensions recorded. The analysis included a large sample of 423 Portuguese participants (151 youth basketball athletes and 272 parents) who completed the Parental Behaviors in Sports (PBSP) questionnaire. The PBSP comprises a version for parents and a version for athletes, composed of five dimensions: (1) sports support, (2) competition attendance, (3) technical influence, (4) performance pressure, and (5) sports expectations. No significant statistical differences regarding PBSP dimensions were observed between boys and girls in the athletes’ group. However, in the parents’ group, fathers scored significantly lower than mothers in sports expectations (*p* = 0.001). Differences between athletes’ and parents’ perceptions were evident in competition attendance (*p* = 0.023), technical influence (*p* ≤ 0.001), and sports expectations (*p* = 0.023). When analyzing the dyads of boys–fathers and girls–mothers, significant differences were found for technical influence (*p* = 0.035), performance pressure (*p* = 0.020), and sports expectations (*p* < 0.001) between boys and fathers. Among girls and mothers, differences were perceived exclusively for technical influence (*p* = 0.005). Finally, technical influence correlated significantly with performance pressure (r = 0.351, *p* < 0.001) and sports expectations (r = 0.367, *p* < 0.001). These findings demonstrate the crucial importance for sports researchers and practitioners to consider differences in perceptions based on sex and role (athlete or parent) when designing and implementing parental educational programs.

## 1. Introduction

Parental involvement in youth sports has become relevant in scientific research, particularly since parents started to become increasingly aware of their role in children’s development in sports [1]. Furthermore, previous studies have reported that parental involvement has implications for children’s enjoyment, motivation, development, and long-term involvement in sports [2,3,4].

According to the literature, parental involvement is the time, energy, and money parents invest in their child’s sports participation [5,6]. This includes transportation, attending practices and games, giving instructional feedback, and making financial investments in sports equipment [5,6]. Based on this, parents have gained a highly visible role in youth sports, and their specific behavior can positively or negatively influence their children [5]. For instance, a study conducted among 341 young athletes has concluded that parents’ directive behaviors and pressure were positively associated with pre-competitive anxiety [4]. In contrast, through a qualitative study that included 55 young athletes, the authors described that participants often reported greater sports enjoyment when they perceived that their parents were positively involved and pleased with their participation [7].

A balanced level of parental participation can promote youth satisfaction with their sport and enhance development through sports. Besides, family dynamics can also influence skill development since parents play a crucial role in encouraging sports participation [8]. However, excessive pressure may lead to stress, reduced performance, and even dropout [9], compromising children’s long-term involvement in sports.

Besides, research has also explained differences in parental involvement and young athletes’ perceptions based on sex. A previous study analyzing parental involvement in youth basketball revealed that fathers were more present in sports participation logistics, while mothers were more interventive in emotional involvement [10]. In another study, which included both team and individual sports young athletes, the findings illustrate that mothers tend to view themselves primarily as sources of praise and understanding, while fathers are more likely to provide directive guidance than mothers [11,12]. Differences between sex perspectives are not exclusive to parents. Among young tennis athletes, praise and understanding were negatively related to anxiety for female athletes only [4]. In another investigation, male athletes indicated higher levels of parental pressure compared to their female peers [13]. Thus, sex emerges as a key variable to be considered while examining parental involvement in the sports practices of their children.

On the other hand, the literature has shown a gap between athletes’ and parents’ perceptions concerning parental involvement practices. In a sample of 352 parents and 256 young athletes, moderate concordance was observed in athletes’ and parents’ perspectives on parental practices [14]. Besides, the differences in perceptions of parental practices between athletes and parents also varied according to parents’ sex [14]. The same trend was found when examining the association of multiple process, person, and context factors with parents’ involvement in sports among 201 athletes aged between 11 and 13 years [12].

Although sex differences in parental involvement have been investigated previously, only a few studies that systematically analyzed athletes’ and parents’ perspectives exist so far [10,12,14]. Moreover, only one study on this important topic has been conducted in youth basketball [10]. Therefore, to address these major gaps in the literature, the aims of this study are twofold: (1) to examine potential differences in athletes’ and parents’ perceptions of parental involvement practices in youth basketball, (2) to verify possible differences between athletes’ and parents’ perceptions based on sex, and (3) to analyze the relationship between the different perceptions dimensions recorded.

## 2. Materials and Methods

### 2.1. Participants

This study included 423 Portuguese participants: 151 youth basketball athletes (aged 14.5 ± 2.3 years, 31.1% males) and 272 parents (aged 46.3 ± 6.7 years, 38.6% males). To participate in this study, the following inclusion criteria were defined: (a) youth basketball players registered in the Portuguese Basketball Federation, (b) ages ranging between 11 and 18 years, and (c) parents of youth basketball players within the age range previously defined. After data collection, a code number was given to each participant to ensure anonymous data analysis.

Sample calculations were conducted a priori using the G*Power 3.1 software [15,16]. A Mann–Whitney U test indicated a total sample size of 244 participants (122 in each group) to achieve 95% power to detect an interaction effect size of 0.5 at the 0.05 significance level. Then, a correlation test suggested a total sample size of 115 participants to achieve 95% power to detect an interaction effect size of 0.5 at the 0.05 significance level.

### 2.2. Instruments

The Parental Behaviors in Sports (PBSP) questionnaire [17] assessed the relationship between parents and youth athletes regarding their sports participation. The questionnaire comprises a version for parents and a version for athletes, composed of five dimensions: (1) sports support, (2) competition attendance, (3) technical influence, (4) performance pressure, and (5) sports expectations. The dimension scores are determined by analyzing 18 questions (Table 1), and the total score of each dimension can range between 1 and 5. Each question is scored using a five-point Likert-type scale ranging from never (1) to always (5). In the athletes’ questionnaire version, youngsters must score each question for their mother and father.

### 2.3. Procedures

The questionnaires were available online through Google Forms for two months (20 July 2024 until 30 September 2024). The link was shared by email among national basketball clubs and associations. The estimated time for completing the questionnaire was around 10 min [17]. All the returning data were exported and organized into Excel files for data analysis. The procedures implemented in this study were previously approved by the Ethics Committee of the University of Madeira (Reference 137/CEUMA/2024, 16 July 2024), and participation was voluntary. Informed consent was obtained from all participants, and in the case of minors, informed consent was also obtained from their respective legal guardians.

### 2.4. Statistical Analysis

Descriptive statistics are presented as median values and were used to explore athletes’ and parents’ responses to the PBSP questionnaire. The Mann–Whitney U test was conducted to compare the results within and between groups based on sex. Afterward, the relationship between the PBSP questionnaire dimensions scores and participants’ sex was evaluated using the Spearman Rho correlation. The strength of the correlation was interpreted as follows [18]: small (r = 0.10 to 0.29), medium (r = 0.30 to 0.49), and large (r ≥ 0.50). The statistical analysis and illustration procedures were conducted using IBM SPSS Statistics 29.0 (SPSS Inc., Chicago, IL, USA) and GraphPad Prism (version 10, GraphPad Software, San Diego, CA, USA). The significance level was set at 5%.

## 3. Results

**Figure 1:**
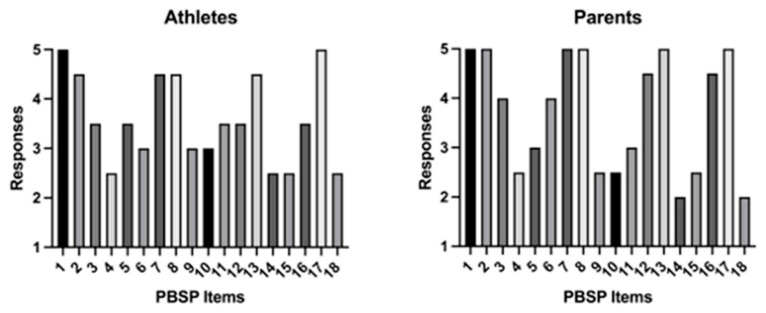
Athletes’ and parents’ responses (median values) to PBSP items.

Figure 1 illustrates athletes’ and parents’ responses (median values) to each question of the PBSP questionnaire, respectively. Overall, differences between groups were observed in items related to performance pressure (questions 9, 14, and 18), technical influence (questions 3, 6, and 11), sports expectations (questions 5 and 10), sports support (question 8), and competition attendance (questions 2 and 7).

**Figure 2:**
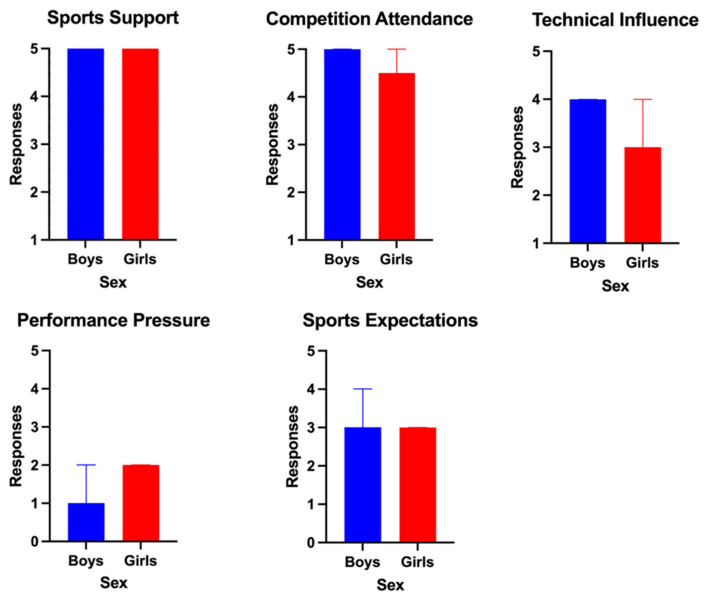
Comparison of the PBSP scores according to sex in the athletes’ group.

**Figure 3:**
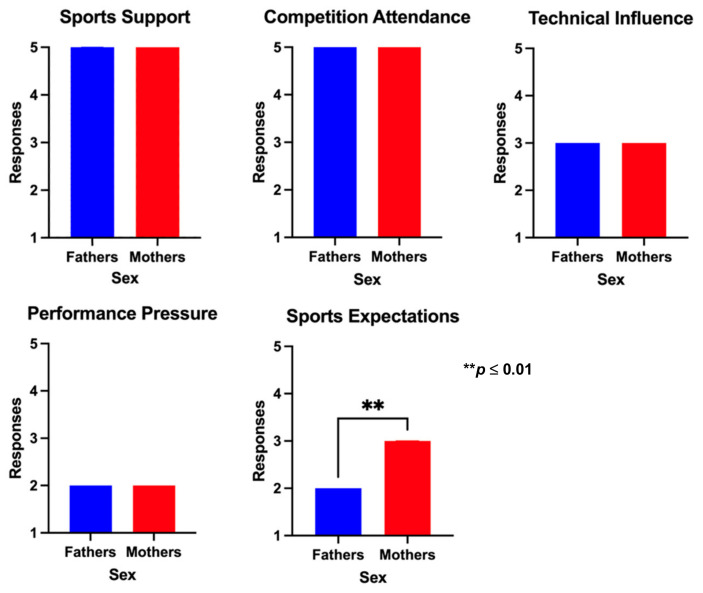
Comparison of the PBSP scores according to sex in the parents’ group.

The comparison based on sex in the scores obtained in each PBSP questionnaire dimension is resumed in Figure 2 and Figure 3 for athletes and parents, respectively. No significant statistical differences were observed between boys and girls. However, boys presented higher median scores in the competition attendance and technical influence and lower median results in performance pressure than girls. In the parents’ group, results were very similar between fathers and mothers, except for the sports expectations dimension, where fathers scored significantly lower than mothers (*p* = 0.001).

**Figure 4:**
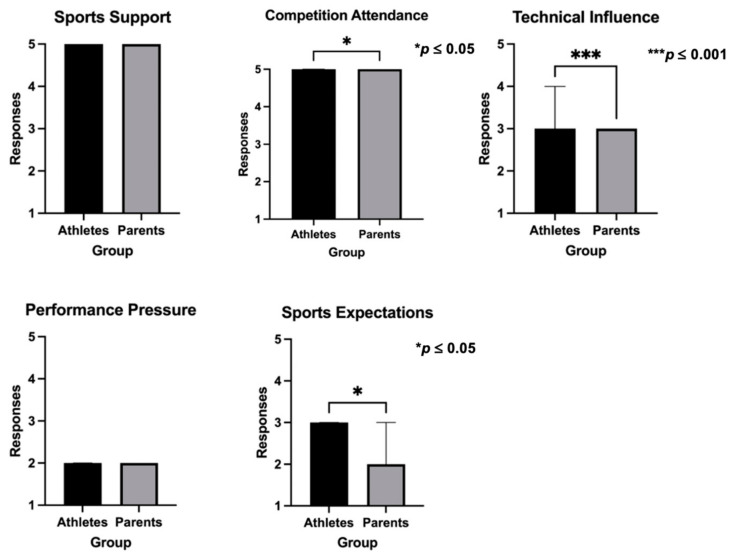
Comparison of the PBSP scores between athletes and parents.

Figure 4 compares athletes’ and parents’ groups on the scores recorded for the PBSP questionnaire dimensions. Significant statistical differences were found for competition attendance (*p* = 0.023), technical influence (*p* ≤ 0.001), and sports expectations (*p* = 0.023). The analysis of the median values for each group showed that parents scored higher in competition attendance, and lower in technical influence and sports expectations.

**Figure 5:**
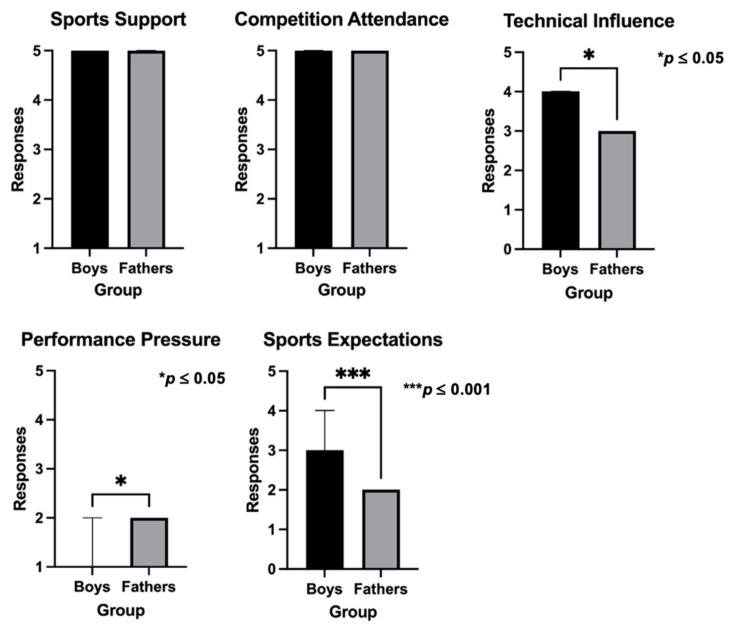
Comparison of the PBSP scores between boys and fathers.

**Figure 6:**
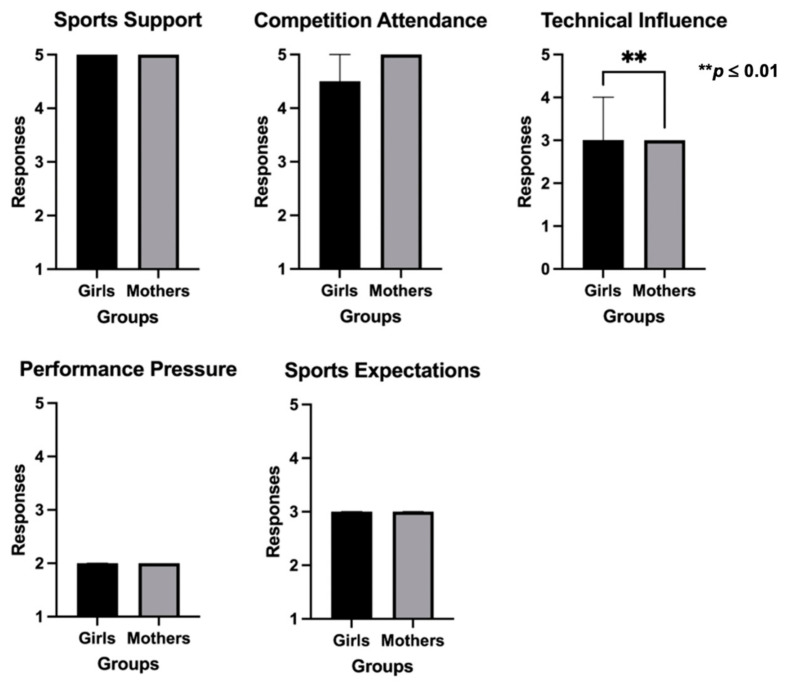
Comparison of the PBSP scores between girls and mothers.

Figure 5 and Figure 6 present the analysis of the dyads of boys–fathers and girls–mothers, respectively. When analyzing data between boys and fathers, boys presented significantly higher scores in technical influence (*p* = 0.035) and sports expectations (*p* < 0.001), and lower scores in performance pressure (*p* = 0.020), than fathers. The same analysis between girls and mothers showed significant statistical differences exclusively regarding technical influence (*p* = 0.005), with girls reporting higher values than mothers.

Finally, when assessing the relationship between the PBSP questionnaire dimensions among all participants (Table 2), the strongest correlations were found between technical influence and performance pressure (r = 0.351, *p* < 0.001, medium), and between technical influence and sports expectations (r = 0.367, *p* < 0.001, medium). A significant medium correlation was also observed between sports support and competition attendance (r = 0.326, *p* < 0.001).

## 4. Discussion

This study examined athletes’ and parents’ perceptions of parental involvement practices in youth basketball and included an analysis of parental practices perspectives based on sex. Among the athletes’ group, although boys presented higher median scores in two dimensions of the PBSP questionnaire (competition attendance and technical influence) and lower scores in performance pressure compared to girls, no significant differences were revealed. However, the comparison within the parents’ group showed that fathers scored substantially lower in sports expectations than mothers. The analysis of both groups’ responses (athletes vs. parents) indicated significant differences in three dimensions assessed in the PBSP questionnaire (competition attendance, technical influence, and sports expectations). When comparing the dyads of boys–fathers and girls–mothers, substantial variation in technical influence, performance pressure, and sports expectations (boys–fathers), as well as technical influence (girls–mothers), were identified. Finally, significant positive correlations were observed between several PBSP questionnaire dimensions, with the strongest one being found between technical influence and sports expectations.

In the athletes’ group, boys scored higher in competition attendance and technical influence than girls. Competition attendance refers to parents’ presence in competition moments, while technical influence represents the tendency of parents to provide advice or suggestions to improve children’s performance [17]. A previous study, developed in male youth soccer, examined the parents’ verbal reactions to competitions, finding a continuous evolution of more supportive (i.e., praise, encouragement) to more controlling comments (i.e., performance feedback, instruction) [19]. Another piece of research on male youth soccer reported that the athletes’ perception of parents’ active involvement and directive behavior were higher than desired [20]. In the current study, it seems that male athletes perceived higher participation in competition and greater technical guidance by their parents than girls. In contrast, performance pressure by their parents seems to be more impactful among girls than boys. The literature has widely described the underrepresentation of girls in sports and the diverse range of barriers associated with women’s and girls’ sports participation, such as attitudinal inequalities [21]. For instance, in a study aiming to explore the relationship between parents’ beliefs and youth sports participation, the results indicated that parents valued sports slightly more for their sons than their daughters, both regarding ideology and financial investment [22]. Moreover, socially constructed phenomena play a significant role in sports. For instance, societal expectations tend to encourage male participation in sports, while female involvement is often not similarly supported [23]. Consequently, there is a cultural misalignment between girls and sports [22], which might enhance their perception of pressure to perform, including in the view of their parents. Besides, the literature has advocated that children report greater athletic competence and intrinsic motivation when they frequently receive positive feedback from their parents and perceive their parents’ beliefs about their abilities as supportive and encouraging [5]. Therefore, to combat the adverse feelings of performance pressure, positive feedback is crucial from parents and should be reinforced among girls.

Interestingly, fathers scored significantly lower in sports expectations than mothers. The existence of biological and psychosocial sex differences is well-established in the scientific literature [24]. Women are described as more sensitive and childcare-driven, while men tend to present more leadership and aggressiveness characteristics [25]. Since the sports expectations dimension refers to the parents’ positive perspective regarding their children’s future in sports [17], the higher scores presented by mothers might reflect their positive support concerning children’s long-term involvement in sports. Thus, sports expectations should not be exclusively interpreted as their intention to see their children attain elite levels in the future.

When comparing athletes’ and parents’ responses, statistically significant differences emerged in three PBSP questionnaire dimensions. Athletes perceived higher technical influence and sports expectations than their parents. Indeed, this gap between athletes’ and parents’ perceptions of parental practices is consistent with previous literature. For instance, an investigation among youth athletes from team sports has reported modest concordance between athletes’ and parents’ parental practices perceptions. Overall, parents reported lower directive behaviors, pressure perceptions, and higher active involvement, praise, and understanding perceptions than athletes [14]. The exact modest correspondence between athletes’ and parents’ perspectives on parental involvement was reported in 201 participants [12]. The current findings are consistent with previous work indicating that children and parents often have differing perceptions of each other’s behaviors [26].

The assessment of the dyads of boys–fathers and girls–mothers also showed a significant variation in various PBSP questionnaire dimensions. More specifically, both boys and girls perceived higher technical influence than fathers and mothers. Boys also perceived higher sports expectations and lower performance pressure than fathers. The literature investigating the contrast between boys–fathers and girls–mothers’ dyads is still scarce. However, in a previous study on this topic, the results indicated that boys’ perspective on pressure is typically higher than that of their fathers, which is consistent with the results of the current study. In comparison, girls’ perspectives on active involvement are lower than those of their mothers [14], which might include their perception of technical influence and justify the current results. These data reinforce the gap between groups’ perceptions, with parents expressing more positive beliefs regarding their role than athletes. According to research, positive parental involvement occurs when parents actively seek to understand their children’s sporting experiences [2]. Also, it is further suggested that parents should work towards establishing and communicating shared goals, foster an emotionally supportive environment, and practice positive behaviors during competitions [2]. This is crucial to secure psychological stability, avoid demotivation and, consequently, sports dropout [3,4]. Therefore, the need for mutual dialogue between youngsters and their parents to share emotions and feelings should be underlined. Communication serves both to strengthen family bonds and to facilitate mutual evaluation among family members. Through effective communication, children and parents can recognize and nurture their potential and talents in the context of sports [27].

In the meantime, when evaluating the correlations between the PBSP questionnaire dimensions, the strongest relationships (positive) were found between technical influence, performance pressure, and sports expectations. Thus, parental education programs targeting youth sports should emphasize the adverse role of excessive technical feedback since it enhances children’s perception of pressure, which can lead to decreased enjoyment and increased anxiety [28]. On the other hand, it is worth noting that sports expectations presented a significant and positive relationship with all the other dimensions assessed. This suggests that parents’ support, presence, and intervention are highly related to their expectations of athletes’ long-term sports involvement. Scientific evidence has emphasized that athletes want their parents to be supportive and involved in their sporting experience [29,30]. However, excessive pressure, and technical and tactical advice, are associated with demotivation and less enjoyment [30]. On the other hand, sports expectations should not be seen by parents as future high-level achievements but as including the athletes’ development through long-term involvement in sports.

The current study presents some limitations that are worth mentioning. First, all participants were Portuguese, and their experiences might differ from youngsters involved in other countries’ sporting realities and cultures. To our knowledge, this is the first study analyzing parental involvement in Portuguese youth sports, including athletes’ and parents’ perspectives. However, in a previous study conducted among Portuguese youth handballers, athletes reported exaggerated pressure from their parents and the perception that their parents get angry when the team results are not desired [31]. Although cultural specificity might affect family-oriented sports culture, there seems to be a trend for discrepancies between athletes’ and parents’ perceptions, particularly regarding technical and tactical feedback, which is corroborated by studies conducted in different countries (Portugal, Canada, Italy) [19,20,31]. Secondly, this study focused on youth basketball at a particular time, and the results should not be extrapolated to other sports, or to previous and future athlete–parent relationships. Future research on long-term parent–athlete relationships could provide a more in-depth analysis of athletes–parents dyads. Finally, it should be noted that this study focused on quantitative analysis, while a qualitative approach based on observations and interviews could provide more detailed data on the variables assessed, as well as on other variables of interest for parental behaviors, such as lifestyles.

Nevertheless, the present study summarizes important results on parental involvement behaviors by considering both the athletes’ and parents’ perspectives, whereas most of the previous research has been mainly focused on the athletes’ perceptions. The differences in perceptions among groups suggest the need to establish dialogue and share perspectives to avoid adverse effects of parental practices that might influence athletes’ motivation and long-term sports participation.

## 5. Conclusions

Parental support that does not lead to excessive technical and tactical advice can foster positive sports experiences. Considering the positive and significant relationship established between technical influence and performance pressure shown in this study, parents should be aware that excessive technical feedback might adversely affect young athletes’ performance by enhancing their perception of pressure. Negative parental involvement can significantly affect children’s psychological responses (i.e., feelings of anxiety, stress, and demotivation), leading to long-term repercussions regarding sports practice. Sports parenting expertise involves parents acquiring knowledge and applying a variety of intrapersonal (i.e., emotional intelligence, coping, implicit beliefs) and interpersonal (i.e., listening, attending, empathy, giving constructive feedback) skills to support their child effectively. On the other hand, this study reinforces differences in perceptions based on sex in both groups, which might be related to psychosocial characteristics, such as the social roles associated with gender and the limited sports participation opportunities that females experience. Overall, the current study brings important insights for sports researchers and practitioners to consider differences in perceptions based on sex and role (athlete or parent) when designing and implementing parental educational programs. Future research should emphasize the analysis of long-term athletes–parents dyads and include qualitative methods to provide more detailed insights into athletes’ and parents’ perceptions.

## Figures and Tables

**Table 1:** Dimensions and classification of the Parental Behaviors in Sports Questionnaire.

Dimensions	Items	
1. Sports support (4 items)	1, 8, 12, 17 Total score = 1–5	Parental support, satisfaction, and interest in their child’s sports activity.
2. Competition attendance (3 items)	2, 7, 13 Total score = 1–5	Parents’ presence at their child’s competitions.
3. Technical influence (4 items)	3, 6, 11, 16 Total score = 1–5	Tendency of parents to provide advice or suggestions on how their child can improve technical skills and how they should train and/or compete.
4. Performance pressure (4 items)	4, 9, 14, 18 Total score = 1–5	Negative parental behaviors towards poor performance in competitions and unfavorable sports results of their child.
5. Sports expectations (3 items)	5, 10, 15 Total score = 1–5	Parents’ positive expectations about their child’s sports future.
	Total items = 18	

**Table 2:** Correlation coefficients among the PBSP questionnaire dimensions, including all participants.

	D1.	D2.	D3.	D4.	D5.
D1.	-	0.326 **	0.215 **	0.008	0.173 **
D2.		-	0.241 **	0.049	0.096 *
D3.			-	0.351 **	0.367 **
D4.				-	0.250 **
D5.					-

D1. Sports support; D2. Competition attendance; D3. Technical influence; D4. Performance pressure; D5. Sports expectations; * *p* ≤ 0.05; ** *p* ≤ 0.01.

## Data Availability

The data presented in the current study are available upon request from the corresponding author.
